# The access and waiting-time standard for first-episode psychosis: an opportunity for identification and treatment of psychosis risk states?

**DOI:** 10.1192/pb.bp.116.054254

**Published:** 2017-02

**Authors:** Richard Whale, Andrew Thompson, Rick Fraser

**Affiliations:** 1 Early Intervention Service, Sussex Partnership NHS Foundation Trust, UK; 2 Brighton and Sussex Medical School, UK; 3 Division of Mental Health and Wellbeing, Warwick Medical School, University of Warwick, Coventry, UK; 4 North Warwickshire Early Intervention in Psychosis Service, Coventry and Warwickshire Partnership NHS Trust, UK

## Abstract

Expansion of early intervention services to identify and clinically manage at-risk mental state for psychosis has been recently commissioned by NHS England. Although this is a welcome development for preventive psychiatry, further clarity is required on thresholds for definition of such risk states and their ability to predict subsequent outcomes. Intervention studies for these risk states have demonstrated that a variety of interventions, including those with fewer adverse effects than antipsychotic medication, may potentially be effective but they should be interpreted with caution.

With the advent of the new access and waiting-time standard for first-episode psychosis published by NHS England in February 2015,^1^ there is now a definite move to adopt service models aimed at preventing transition to psychosis in vulnerable individuals, as originally developed in 1994 by the Personal Assistance and Crisis Evaluation clinic in Melbourne.^2^ There is an expectation that early intervention in psychosis services will now also offer interventions for at-risk mental state for psychosis (ARMS), based on our evolving understanding of best practice in this area.

This move is exciting for a number of reasons. It represents a commitment from the Government to support mental health service development and reform, especially preventive approaches, at a time when many services are experiencing cuts. Cost-effectiveness of ARMS services has been demonstrated.^3^ Second, as a treatment paradigm the preventive strategy represents a possibility that we can alter the trajectory of a potentially serious condition and improve outcomes in all domains, including symptoms and functioning. Third, we may be able to use, at an earlier stage of illness, more benign treatments that are potentially less costly, less stigmatising and better tolerated.^2^ This preventive model also represents an opportunity to broaden treatment paradigms within mental health, not just for psychosis but for other disorders, fitting perfectly with another current health development strategy – low-stigma, accessible and responsive youth mental health services. Debate continues as to whether such services are appropriately placed within established early intervention for psychosis or whether new, dedicated teams with a more public health emphasis should be created. However, existing services have expertise in both defining first-episode psychosis thresholds and offering relevant clinical support packages for both ARMS and first-episode psychosis.^4^

The criteria commonly used in the UK for ARMS depend on the presenting clinical features, relative functional impairment and help-seeking.^2^ Consistent quantification of distress relating to these features is currently lacking. It also remains unclear how these clinical risk features differ from more widespread psychotic phenomena in the general population. Psychotic experiences in non-help-seeking populations appear relatively common, affecting about 5%,^5^ and higher in child and adolescent samples;^6^ there is apparent sharing of aetiological risk factors with schizophrenia. Clinical outcomes of this non-help-seeking group are unknown. Psychosis transition threshold is commonly defined by three Positive and Negative Syndrome Scale items (delusions, hallucinations or conceptual disorganisation) achieving adequate severity for at least 7 days,^7^ but such psychosis thresholds are not without controversy.^8^ The large majority of those identified as ARMS do not cross this severity threshold within 3 years of follow-up, although many remain functionally impaired or develop other disorders.^2^ Whether other transition criteria, or modifications of existing criteria, are better able to predict longer-term outcome remains to be established. The reliability of identifying such thresholds in clinical practice is also less than in research settings,^9^ despite using widely available tools.^2^ This is further complicated by concurrent substance misuse, common in such clinical populations. However, the definition and adoption of such thresholds is clearly necessary to educate clinicians, decide when to appropriately intervene and support research. The complexity of the psychosis sub-syndrome groups (including individuals with a family history of psychotic illness, those with schizotypal disorder or the attenuated psychosis syndrome, those with brief limited intermittent notable severity psychotic episodes and those help-seeking or not) and their undetermined probable outcomes may lead to services primarily adopting a more discrete threshold for inception, such as the DSM-5 research-appendix-defined attenuated psychosis syndrome, which has marked clinical overlap with ARMS-defined populations.^9^

Without clear diagnostic robustness of a condition, and with a wide variation in clinical outcome, interpretation of intervention studies is problematic. Initially, randomised studies of diverse interventions for operationally defined ARMS (termed ultra high risk for psychosis) seemed to show similar beneficial effects *v.* control. Reviews pooling outcomes of these studies clearly advocated intervention.^7,10^ More recent randomised studies have demonstrated less clear benefits over control than earlier studies, as is often seen in health research (arguably ‘active’ controls were used in many of these studies). Primary intervention recommendations of supportive counselling/case management for this clinical group have emerged, as previously used as a control intervention. Several factors will need to be considered, with future investigations including previous low sample size due to recruitment problems, use of robust and consistent thresholds for group inclusion, and transition to psychosis to reduce heterogeneity of outcome, consistent inclusion of functional outcomes, translation of findings to usual clinical care (away from research clinics), ensuring timely publication of results and the importance of replication of existing findings.

While considerable progress has been made in this area, we remain at the early stages of defining a risk syndrome for psychosis. The currently adopted clinical threshold for ARMS seems to be a valid construct to identify clinical need but the heterogeneity of subsequent clinical outcomes is wide. Specific interventions for ARMS are unclear, aside from those for commonly identified comorbidities (such as anxiety, depression and substance misuse). Intervention studies to date highlight the importance of methodological rigour and consistency of diagnostic thresholds used, to which end the DSM-5 attenuated psychosis syndrome may be a positive step.^9^ Biological models for psychosis risk need replication, clinical validation and combining with clinical markers in larger, longitudinal studies to enhance risk determination.^2,11,12^

Despite these caveats, this field of study represents an important advance in the development of preventive psychiatry. The current move to incorporate earlier psychosis states in more widespread clinical services, with appropriate threshold definition and outcome monitoring, may also have important societal impact.
